# Ribosomal Protein Cluster Organization in Asgard Archaea

**DOI:** 10.1155/2023/5512414

**Published:** 2023-09-29

**Authors:** Madhan R. Tirumalai, Raghavan V. Sivaraman, Layla A. Kutty, Eric L. Song, George E. Fox

**Affiliations:** ^1^Department of Biology and Biochemistry, University of Houston, Houston, TX 77204-5001, USA; ^2^Silver Lab, Wyss Institute for Biologically Inspired Engineering, Boston, MA, USA; ^3^University of Texas at Austin, Austin, TX, USA

## Abstract

It has been proposed that the superphylum of Asgard Archaea may represent a historical link between the Archaea and Eukarya. Following the discovery of the Archaea, it was soon appreciated that archaeal ribosomes were more similar to those of Eukarya rather than Bacteria. Coupled with other eukaryotic-like features, it has been suggested that the Asgard Archaea may be directly linked to eukaryotes. However, the genomes of Bacteria and non-Asgard Archaea generally organize ribosome-related genes into clusters that likely function as operons. In contrast, eukaryotes typically do not employ an operon strategy. To gain further insight into conservation of the r-protein genes, the genome order of conserved ribosomal protein (r-protein) coding genes was identified in 17 Asgard genomes (thirteen complete genomes and four genomes with less than 20 contigs) and compared with those found previously in non-Asgard archaeal and bacterial genomes. A universal core of two clusters of 14 and 4 cooccurring r-proteins, respectively, was identified in both the Asgard and non-Asgard Archaea. The equivalent genes in the *E. coli* version of the cluster are found in the *S10* and *spc* operons. The large cluster of 14 r-protein genes (*uS19-uL22-uS3-uL29-uS17* from the *S10* operon and *uL14-uL24-uL5-uS14-uS8-uL6-uL18-uS5-uL30-uL15* from the *spc* operon) occurs as a complete set in the genomes of thirteen Asgard genomes (five Lokiarchaeotes, three Heimdallarchaeotes, one Odinarchaeote, and four Thorarchaeotes). Four less conserved clusters with partial bacterial equivalents were found in the Asgard. These were the *L30e* (*str* operon in Bacteria) cluster, the *L18e* (*alpha* operon in Bacteria) cluster, the *S24e-S27ae-rpoE1* cluster, and the *L31e*, *L12..L1* cluster. Finally, a new cluster referred to as *L7ae* was identified. In many cases, r-protein gene clusters/operons are less conserved in their organization in the Asgard group than in other Archaea. If this is generally true for nonribosomal gene clusters, the results may have implications for the history of genome organization. In particular, there may have been an early transition to or from the operon approach to genome organization. Other nonribosomal cellular features may support different relationships. For this reason, it may be important to consider ribosome features separately.

## 1. Introduction

Ever since the initial discovery of Archaea by Woese and Fox [1], the number of recognized lineages comprising the Archaea domain has rapidly increased. Currently, five superphyla (and 35-38 phyla) of Archaea are described in the NCBI database [2–4]. Of these, the recently discovered Asgard include several phyla and are the most diverse [5, 6]. To emphasize this, they have been referred to as a “superphylum” [7].

Amongst the three domains, there are approximately 102 recognized ribosomal protein (r-protein) families [8–11]. Essentially universal amongst these are seventeen large subunit and nineteen small subunit r-proteins. Many of these 36 r-proteins likely had an ancient origin possibly before the last universal common ancestor (LUCA) [10–16]. However, this conclusion is dependent on how the gene content of LUCA is identified. At least eight of these genes are found in multiple versions of LUCA (Rivas personal communication). The Archaea and the Eukarya also share eleven large subunit (LSU) r-proteins and twenty small subunit (SSU) r-proteins. However, neither group shares additional r-proteins with the Bacteria. For this reason, it has been hypothesized [17, 18] that the eukaryotic translation system originated from an earlier archaeal version and was subsequently expanded.

In Bacteria, approximately 32 r-proteins from both subunits and two translation-related proteins are grouped into seven well-studied clusters. They are, viz., *alpha*, *beta*/*L10*, *L11*, *S10*, *S20*, *str*, and the *spc* clusters [19, 20]. In *E. coli* and other well-studied organisms, these clusters have been experimentally shown to be operons [21]. In general, we will refer to them as clusters with the implication that many of them could likely be operons. The *S10* and the *spc* operons (clusters) are the largest, each typically encoding ten-twelve r-proteins [21, 22]. Gene expression when known is frequently regulated by one of the r-protein components within the cluster. Besides these clusters, there are several smaller clusters as well. The r-protein clusters are widely conserved amongst bacterial species and remnants, and Bacteria-like clusters are also found in some chloroplasts and mitochondria [23]. However, it is not clear if the clusters seen in mitochondria are functional. If they are not, then their presence or absence is likely a mere reflection of their nonessentiality.

The universality of r-protein gene clusters is often linked with a long history of their association. They also represent ancient/regulatory relationships, with implications for early gene clusters in mini chromosomes. Eukaryotic transcription usually involves a single transcript. Thus, eukaryotic operons/clusters of genes are restricted to few organisms [24, 25]. While eukaryotes do have meaningful gene clusters, these clusters do not include r-protein genes [24, 26–29].

Earlier studies of non-Asgard Archaea showed that the universal r-protein genes are in clusters similar or identical to those found in Bacteria [20]. While three archaeal r-protein genes *S4e*, *L32e*, and *L19e* were found associated with the archaeal version of the *spc* operon, the nonuniversal archaeal r-protein gene *L18e* was part of the conserved *L13* cluster. 17 r-protein genes were part of one of the ten previously unrecognized gene clusters. These clusters were found to be associated with genes involved in the initiation of protein synthesis, transcription, or other cellular processes. Since such associations in the universal clusters could not be found, it was posited that the ribosome had its own independent line of evolution [20].

It has been clear since their initial discovery that archaeal ribosomes more closely resemble those of Eukarya than Bacteria as has been documented in detail [30–32]. However, the non-Asgard Archaea have not been shown to have any strong specific relationship to Eukarya, and the matter was not initially aggressively pursued. Efforts to understand the relationship between the domains of life were rekindled when it was discovered that the then newly discovered Asgard Archaea have many nonribosomal features that are shared with Eukarya [7, 31, 32]. This has fostered the hypothesis that Eukarya may have descended from the Archaea [33]. Asgard Archaea were proposed to represent a historical link or bridge between Archaea and Eukarya [34–41]. A recent report has suggested that the genomic material is condensed and spatially distinct from the riboplasm within certain Loki- and Heimdallarchaeota cells, as further proof of the role of Asgard Archaea in eukaryogenesis [42]. Amongst the Asgard group, the Heimdallarchaeota have been proposed to be the closest to the Eukarya [43, 44]. Other analyses based on multiple gene sequences placed the Asgard between the TACK group of Archaea and Eukarya [7, 36, 40]. In contrast, one branch of the Asgard, namely, the Lokiarchaeota, and their close relatives were proposed to be closer to the Euryarchaeota, than to Eukarya [45].

Genes that are translated together frequently share similar origins, physical interactions, functions, or regulatory mechanism(s) [46]. The arrangement of genes in genomes is thus a window to understand how organisms are related. Assuming there was a universal ancestor of the three domains, it likely had a gene order/arrangement that possibly was a precursor of operons. Its evolutionary origins may have dated back to four billion years ago [47–51]. While some portions of this gene order underwent shuffling over evolutionary timescales [52, 53], the arrangement of some gene clusters continues to be conserved [54]. In particular, the translation machinery and the genes that encode the same are highly conserved. Their association frequently has had a long evolutionary history [55–58]. The origin of the ribosome has been posited to be strongly coupled with the early history and even origins of life [56, 59–63], which is further supported by recent papers by Bose et al. [64] and Bose et al. [65].

In order to gain further insight into the r-protein gene clusters in Asgard Archaea, the genome order of r-protein coding genes was analyzed and compared with non-Asgard archaeal and bacterial genomes.

## 2. Materials and Methods

### 2.1. Retrieval of Sequences of Genomes and Protein Coding Sequences

The data used herein are based on the availability of sequences as of July 2023. The feature table and GenBank/RefSeq sequences of the genomes of Asgard Achaea were obtained from the public databases of the National Center for Biotechnology Information (NCBI) [66–68] and the Integrated Microbial Genomes and Microbiomes (IMG/M) system of the DOE's Joint Genome Institute (JGI) [69, 70]. All the raw genomes and the annotated gene and protein sequences of the same were saved as such and used. When required, r-protein sequences were obtained from the ProteoVision server [71]. Genomes that were not annotated and deposited as raw sequences were excluded.

### 2.2. Mapping of the r-protein Gene Cluster(s)

**Figure 1:**
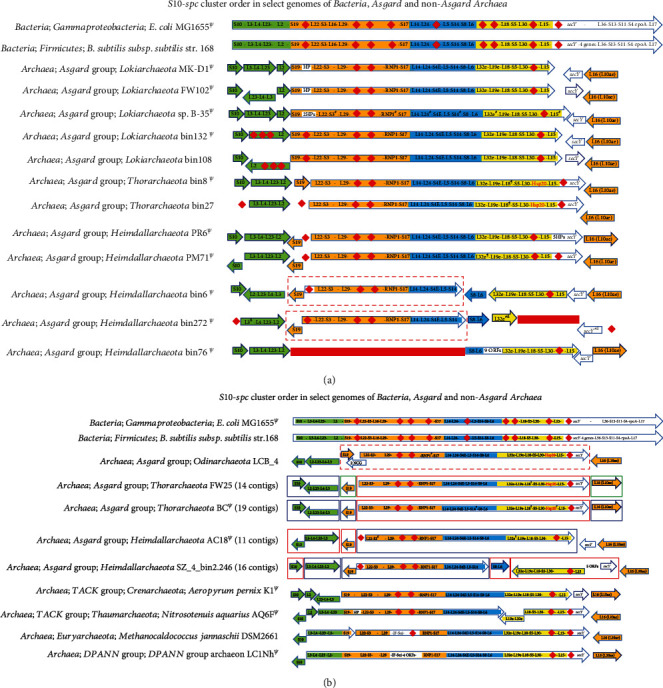
(a) ^*Ψ*^The orientation of all the genes in the entire cluster flipped for aligning with the other genomes. Each arrow represents a different location on a single complete scaffold; adjacent arrows do not indicate their order on the genome; genes within an arrow are contiguous. ^¶^Pseudogene; red diamond and red rectangle represent gene(s) absent in the corresponding genome/location; arrows within dashed red boxes are contiguous; HP: hypothetical protein; RNP1: ribonuclease P protein component 1; ORF: open reading frame/gene; ^#^ORF(s) annotated as HP(s); Hsp20: heat shock protein 20; ^*ß*^partial gene. (b) ^*Ψ*^The orientation of all the genes in the entire cluster flipped for aligning with the other genomes. In *Thorarchaeota* and *Heimdallarchaeota*, each colored (blue, green, or red) box represents a different contig; adjacent arrows do not indicate their order on the genome unless they are within a dashed red box; genes within an arrow are contiguous. ^¶^Pseudogene; red diamond represents gene(s) absent in the corresponding genome/location; arrows within dashed red boxes are contiguous; HP: hypothetical protein; RNP1: ribonuclease P protein component 1; ORF: open reading frame/gene; ^#^ORF(s) annotated as HP(s); Hsp20: heat shock protein 20; ^*ß*^partial gene; IF Sui: protein translation factor SUI1 homolog; NCG: noncluster genes.

**Figure 2:**
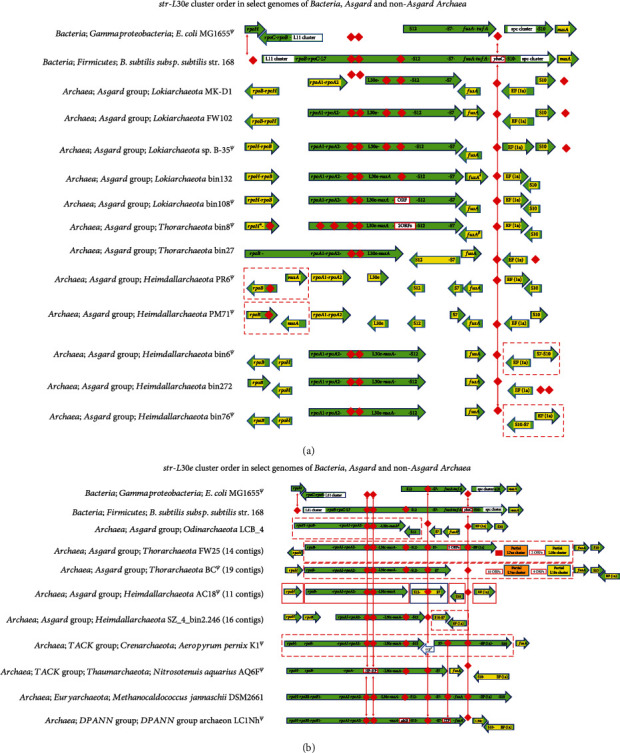
(a) ^*Ψ*^The orientation of all the genes in the entire cluster flipped for aligning with the other genomes. Each arrow represents a different location on the single complete scaffold/genome; adjacent arrows do not indicate their order on the genome; arrows within dashed red boxes are contiguous; genes within an arrow are contiguous; red diamond represents gene(s) absent in the corresponding genome/location. *ybaC*: proline iminopeptidase; HP: hypothetical protein; ^#^ORF(s) annotated as HP(s); gene in yellow color occurs outside the main cluster in that genome; ^*β*^partial gene; ^*α*^duplicate copies of the gene adjacent to each other. (b) ^*Ψ*^The orientation of all the genes in the entire cluster flipped for aligning with the other genomes. Adjacent arrows do not indicate their order on the genome; arrows within dashed red boxes are contiguous; genes within an arrow are contiguous; red diamond and red rectangle represent gene(s) absent in the corresponding genome/location; in Heimdallarchaeote AC18, each colored (blue, green, or red) box represents a different contig; *ybaC*: proline iminopeptidase; HP: hypothetical protein; ^#^ORF(s) annotated as HP(s); genes in yellow color occur outside the main cluster in that genome; ^*β*^partial gene; ^*α*^duplicate copies of the gene adjacent to each other; ccp: crenarchaeal conserved protein; *ubiB*: ubiquinone biosynthesis protein coding gene.

**Figure 3:**
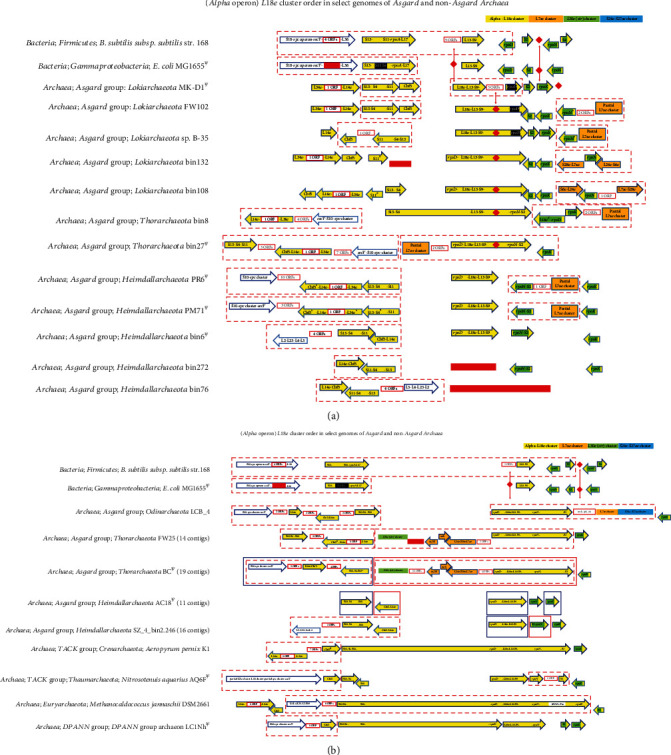
(a) ^*Ψ*^The orientation of all the genes in the entire cluster flipped for aligning with the other genomes. Adjacent arrows do not indicate their order on the genome; genes within an arrow are contiguous; red diamond and red rectangle represent gene(s) absent in the corresponding genome/location; arrows within dashed red boxes are contiguous; genes in white color (black background) are not in the order as found in the other genomes; genes in green color occur outside the main cluster in that genome; ^#^ORF(s) annotated as HP(s); ^¶^pseudogene. (b) ^*Ψ*^The orientation of all the genes in the entire cluster flipped for aligning with the other genomes. Adjacent arrows do not indicate their order on the genome; ^^^begins a contig; genes within an arrow are contiguous; red diamond and red rectangle represent gene(s) absent in the corresponding genome/location; arrows within dashed red boxes are contiguous; in Thorarchaeote BC and Heimdallarchaeotes AC18 and SZ_4_bin2.246, each colored (blue, green, or red) box represents a different contig; genes in white color (black background) are not in the order as found in the other genomes; ^¶^pseudogene; genes in green color occur outside the main cluster in that genome; ^#^ORF(s) annotated as HP(s).

**Figure 4:**
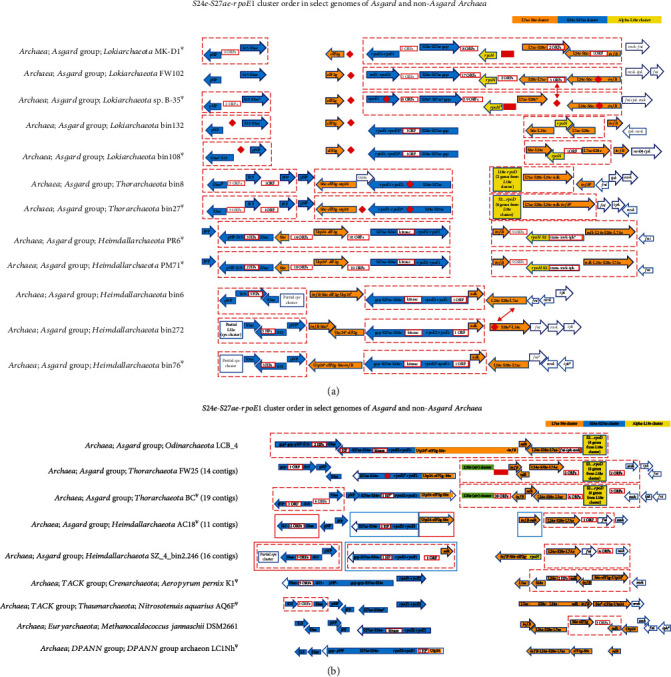
(a) *S24e-S27ae-rpoE1 cluster.*^*Ψ*^The orientation of all the genes in the entire cluster flipped for aligning with the other genomes. Adjacent arrows do not indicate their order on the genome except when they are boxed inside dashed red boxes; genes within an arrow are contiguous; red diamond and red rectangle represent gene(s) absent in corresponding genome/location; arrows within dashed red boxes are contiguous. HP: hypothetical protein; pNP: noncanonical purine NTP pyrophosphatase; InfB: translation initiation factor IF-2; ndk: nucleoside diphosphate kinase; Utp24: 30S proteasome protein; ^#^ORF(s) annotated as HP(s); gcp: bifunctional tRNA threonylcarbamoyladenosine biosynthesis protein; *fni-ipk-mvk*: archaeal lipid pathway genes; ^*ß*^partial gene. (b) *S24e-S27ae-rpoE1 cluster.*^*Ψ*^The orientation of all the genes in the entire cluster flipped for aligning with the other genomes. Adjacent arrows do not indicate their order on the genome except when they are boxed inside dashed red boxes; genes within an arrow are contiguous; red diamond and red rectangle represent gene(s) absent in corresponding genome/location; arrows within dashed red boxes are contiguous; in Heimdallarchaeotes AC18 and SZ_4_bin2.246, each colored (blue, green, or red) box represents a different contig. HP: hypothetical protein; pNP: noncanonical purine NTP pyrophosphatase; InfB: translation initiation factor IF-2; ndk: nucleoside diphosphate kinase; Utp24: 30S proteasome protein; ^#^ORF(s) annotated as HP(s); gcp: bifunctional tRNA threonylcarbamoyladenosine biosynthesis protein; *fni-ipk-mvk*: archaeal lipid pathway genes.

**Figure 5:**
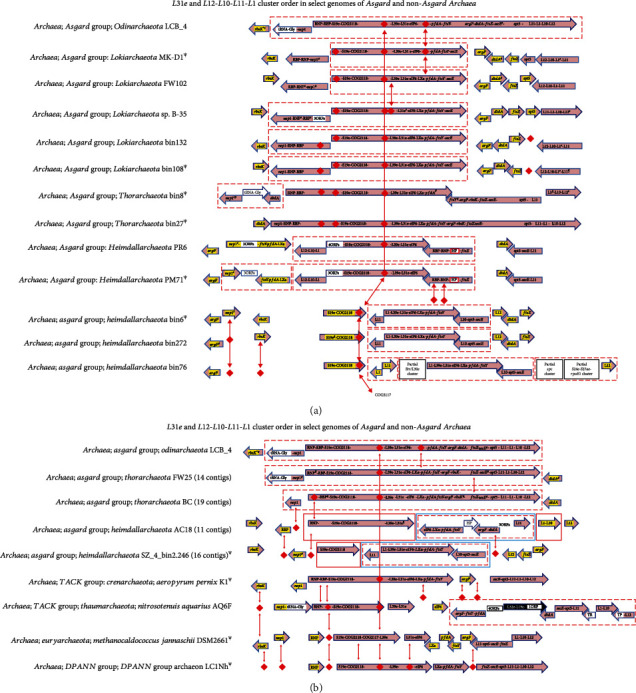
(a) *L31e* and *L12-L10-L11-L1* cluster. ^*Ψ*^The orientation of all the genes in the entire cluster flipped for aligning with the other genomes. Adjacent arrows do not indicate their order on the genome except when they are boxed inside dashed red boxes; genes within an arrow are contiguous; red diamond represents gene(s) absent in the corresponding genome/location; arrows within dashed red boxes are contiguous; genes/ORFs not colored are not the usual components of this cluster. TP: trimeric intracellular cation-selective channel proteinellular cation-selective channel protein; HP: hypothetical protein; ^∗^gene begins or terminates a contig; RNP: ribonuclease P protein component 4; RBP: RNA binding protein; *nep1*: ribosomal RNA small subunit methyltransferase gene; ^#^ORF(s) annotated as HP(s); TR: transcriptional regulator; genes in yellow color occur outside the core/cluster. (b) *L31e* and *L12-L10-L11-L1* cluster. ^*Ψ*^The orientation of all the genes in the entire cluster flipped for aligning with the other genomes. Adjacent arrows do not indicate their order on the genome except when they are boxed inside dashed red boxes; genes within an arrow are contiguous; red diamond represents gene(s) absent in the corresponding genome/location; arrows within dashed red boxes are contiguous; genes/ORFs not colored are not the usual components of this cluster; in the two Heimdallarchaeotes, each colored (blue, green, or red) box represents a different contig. TP: trimeric intracellular cation-selective channel protein; HP: hypothetical protein; ^∗^genes begins or terminates a contig; RNP: ribonuclease P protein component 4; RBP: RNA binding protein; *nep1*: ribosomal RNA small subunit methyltransferase gene; ^#^ORF(s) annotated as HP(s); genes in white (black background) are part of the big *S10-spc* cluster, but occurring with the *secE-spt5-L11* segment in the Thaumarchaeote *N. aquarius* AQ6f; TR: transcriptional regulator; genes in yellow color occur outside the core/cluster.

The feature table of each genome was first checked for the presence/absence of genes in any given cluster. The closest available homologs of those genes that appeared to be missing in a given Asgard genome were used to ascertain the presence/absence of the same using stand-alone blast [72]. Missing genes, which were found to be misannotated as “hypothetical proteins,” were identified using blastX search and/or gene sequence alignment and included in the cluster map(s) (Figures 1–5).

## 3. Results

A total of ~552 *Asgard* genomes grouped under the various phyla that were available on the NCBI server (https://www.ncbi.nlm.nih.gov/datasets/genome/?taxon=1935183) as of July 2023 were examined [36, 73]. Of all the *Asgard* genomes, only thirteen occur as a single complete (scaffold) genome(s) (Table 1). They will henceforth be referred to as MKD1, FW102, B-35, bin132, bin108 (Lokiarchaeota), LCB4 (Odinarchaeota), bin27 and bin8 (Thorarchaeota), PM71, PR6, bin6, bin76, and bin272 (Heimdallarchaeota). The next genomes that are of reasonably good quality (assembled into less than 20 contigs) with annotated genes/proteins are those of the Thorarchaeote strains FW25 (twelve contigs) and BC (nineteen contigs) and the Heimdallarchaeote strains AC18 (eleven contigs) and SZ_4 bin2.246 (sixteen contigs). They will henceforth be referred to as FW25, BC, AC18, and bin2.246, respectively. These genomes provide useful information for some of the clusters of interest but typically not all.

The remaining Asgard genomes each consist of at least twenty contigs and frequently many more. Given the incompleteness of these genomes, the r-protein coding genes in a given cluster were often found in multiple contigs. Some of them, as illustrated in Supplementary Figures 1a-b, either end or begin a contig. The genomes (contigs) of many other genomes have not been annotated into ORFs. Hence, their gene order is not available and therefore was not considered when mapping the clusters. In summary, only the complete genomes and those available with less than twenty contigs, as defined above, were used for the comparative analysis of r-protein gene arrangement. Thus, the choice of non-Asgard archaeal genomes for representation in the figures for comparison is based on the four major groups classified under Archaea [74], viz., the Asgard, the DPANN, the TACK, and the Euryarchaeota [2, 7, 34, 36, 40, 41, 75–78]. Furthermore, the phylogenetic relatedness as described in the literature was also taken into account [7, 34, 37, 39, 41, 79]. *E. coli* and *B. subtilis* were used as representatives of Bacteria.

### 3.1. Big Cluster Comprising Genes from *S10* and *spc*

A core of 14 cooccurring genes (*uL22-uS3-uL29-uS17-uL14-uL24-uL5-uS14-uS8*-*uL6*-*uL18-uS5-uL30-uL15*) belonging to the segment of the *S10*-*spc* cluster is characterized and established as operons in *E. coli* and other Bacteria. In the archaeal (both Asgard and non-Asgard) genomes, this cluster core has 4 additional Archaea-Eukarya-specific genes (*RNP1 (ribonuclease protein 1)*, *S4e*, *L32e*, and *L19e*) in Asgard and non-Asgard genomes (Figures 1(a) and 1(b)). The main core (of 14 genes) was identified as conserved in gene order, arrangement, and genome location in Asgard Archaea, non-Asgard Archaea, and Bacteria (Figure 1). The one exception is in the Thaumarchaeote *N. aquarius* Aq6f (Figure 1(b)). In this genome, a part of this cluster, viz., the segments *L19e-L32e* and *uL18-uS5-uL15*, occurs in the immediate neighborhood of the *L11-L1-L10-L12* cluster.

In Lokiarchaeote sp. B-35, six genes of this cluster (*uS3*, *RNP1*, *uL24*, *uS14*, *L32e*, and *uL15*) are incorrectly annotated as hypothetical proteins. Nevertheless, this core occurs as a complete set in the complete genomes of Asgard Archaea (listed in Table 1) except four Heimdallarchaeote genomes (bin6, bin272, bin76, and bin2.246) (Figures 1(a) and 1(b)).

In the Thorarchaeotes bin8, bin27, FW25, and BC and the Odinarchaeote LCB, a gene for a heat shock protein occurs as an addition to this cluster (Figures 1(a) and 1(b)). A domain analysis using the Conserved Domain Database (CDD) [80] showed that this protein belongs to the family of HSP20 (small heat shock protein IbpA) family (COG0071) involved in posttranslational modification, protein turnover, and chaperones. Acquisition of this gene in these Asgard Archaea could be an unusual instance of horizontal gene transfer occurring within a highly conserved cluster. Additionally, in Thorarchaeotes bin8 and bin27, *uL18* is annotated as a pseudogene.

In the Heimdallarchaeote bin6, the cluster is split into three segments found on different parts of the complete genome. Ten of these genes (*uL22..uS14*) (segment 1) are in one contiguous section, while *uS8-uL6* (segment 2) occurs separately in a second segment. Six genes (*L32e-L19e-uL18-uS5-uL30-uL15*) (segment 3) are in another contiguous section. In bin272, segments 1 and 2 are similarly found separately on the genome. However, only a partial version of the gene *L32e* from segment 3 is present, while the rest of segment 3 is missing. In bin76, segment 1 is missing in entirety, while segments 2 and 3 are present. Additionally, in Lokiarchaeote bin108 and Heimdallarchaeotes bin76 and bin272, 10%, 15%, and 21% of the genes are partial. In the case of the Asgard genomes, which are only represented as contigs, the core occurs as a contiguous set on one contig in FW25, BC, and AC18. Only in the case of Heimdallarchaeote SZ_4_bin2.246 (sixteen contigs), segment 1 is on one contig, while segments 2 and 3 are both at different locations on another contig. This pattern is observed even in the Asgard genomes that consist of more than twenty contigs. In these genomes, the *S10-spc* r-protein cluster organization, despite being found in multiple contigs, is considered indicative of cooccurrence, if the genomes were complete (Supplementary figure 1). In all the non-Asgard Archaea examined, the entire cluster occurs as one contiguous set of genes (with the exception of the Thaumarchaeote *N. aquarius* AQ6f).

### 3.2. Small Cluster of Genes Normally Found in the *S10* Operon

A second smaller cluster comprising the homologs of the most conserved genes of the bacterial *S10* operon/cluster, namely, *uS10-uL3-uL4-uL23-uL2*, occurs independently on the *Asgard* genomes, separate from the rest. This feature is shared by some non-*Asgard* Archaea as well (Figures 1(a) and 1(b)). Part of the small cluster, *uL3-uL4-uL23*, always cooccurs. In contrast, *uS10* is always found to occur independently from this cluster in both the Asgard and non-Asgard Archaea. In bin8, bin27, FW25, and BC (Thorarchaeotes) and PR6, PM71, bin6, bin272, AC18, and SZ_4_bin2.246 (Heimdallarchaeotes), *uL3-uL4-uL23-uL2* occurs as one whole segment. In MKD1, FW102, and B-35 (Lokiarchaeotes), *uL2* occurs independently, separate from the remainder of the small cluster. However, *uL3-uL4-uL23* is entirely missing in bin132 and bin108 (Lokiarchaeotes).

In the bacterial genomes, *E. coli* and *B. subtilis*, the *S10* and *spc* operons (clusters) cooccur as one contiguous cluster of twenty-one genes, including this smaller cluster of five genes. This is then followed by the smaller cluster of *bL36-uS13-uS11-uS4-rpoA-bL17*, which is part of another cluster, namely, the Alpha operon/*L18e* cluster, described below. Thus, in the bacterial genomes, the *S10-spc* operon/cluster is contiguous with a part of the *S13…S9* cluster. *L18e* being Archaea-Eukarya-specific is not found in this segment.

### 3.3. *Str/L30e* Cluster

The core of this cluster is comprised of nine contiguous genes, which is observed to cooccur with the *L11* cluster in *B. subtilis* (Bacteria). In the *E. coli* genome, the cluster is split into two sections. One section comprising *rpoB-rpoC* cooccurs with the *L11* cluster, while the other section is *S12*..*tufA*. Each section is found in different parts of the genome (Figures 2(a) and 2(b)). The equivalent of the *str/L30e* cluster in non-Asgard Archaea (*A. pernix K1* (TACK group; Crenarchaeota), *M. jannaschii* DSM2661 (Euryarchaeota)) and the DPANN group Archaea (Figure 2) has 9-10 genes, viz., *rpoH-rpoB-rpoA-L30e-nusA-S12-S7-(fusA)-EF(1a)-S10*. In the Thaumarchaeote *N. aquarius* AQ6f and DPANN group archaeon LC1Nh, *EF(1a)-S10*fusA occurs independent of this section. In the genomes of the Lokiarchaeotes FW102, B-35, bin132, bin108, and bin8; the Thorarchaeotes FW25 and BC; the Odinarchaeote LCB; and the Heimdallarchaeotes (bin6, bin272, bin76, and bin2.246), a part of this cluster, viz., *rpoA1-rpoA2-L30e-nusA-S12*, occurs as a conserved core. In the other Asgard genomes, this core is split into two parts, comprised of *rpoA1-rpoA2-L30e-nusA* (five genes) and *S12-S7* (two genes), respectively, which are found on different regions of the genome(s). Notable are the complete genomes of the Heimdallarchaeotes PR6 and PM71, in which the entire cluster is dispersed with no conservation of the gene order (Figure 2(a)). Additionally, in the Thorarchaeote genomes FW 25 and BC, the str/L30e cluster cooccurs with a portion each of the *L7ae* (a new cluster described below) and the *L18e* clusters. Despite these two genomes occurring in fourteen and nineteen contigs, respectively, the cluster is fairly contiguous in one contig. The arrangement of the *str/L30e* cluster in the Asgard Thorarchaeota most closely resembles the arrangement found in the non-Asgard Archaea.

### 3.4. Alpha Operon/*L18e* Cluster

This cluster is an operon (*S13-S11-S4-rpoA-L17*) in bacterial genomes and is found immediately downstream of the *S10-spc* operon (Figure 3). In the non-Asgard Archaea, *A. pernix* K1 (TACK group) and *M. jannaschii* DSM 2661 (Euryarchaeota), this cluster is comprised of a contiguous core of eight genes, viz., *S13-S4-S11-rpoD-L18e-L13-S9-rpoN* (Figure 3(b)). Only in *M. jannaschii* DSM 2661, this cluster is in the genomic neighborhood of the *uL3-uL4-uL23-uL2* section of the *S10* cluster.

In the Asgard genomes, the *L18e* cluster is split into two main sections. The first is comprised of *S13-S4-S11*, which, in five Asgard genomes, is found cooccurring with *L34e-L14e-Cbf5* in the genomic neighborhood of the big *S10-spc* cluster (Figures 3(a) and 3(b)). *Cbf5* encodes an RNA-guided pseudouridylation complex pseudouridine synthase subunit. In three Asgard genomes, the *S13-S4-S11* group is in the immediate genomic neighborhood of *uL3-uL4-uL23-uL2*.

The second section is comprised of *rpoD-L18e-L13-S9* (Figures 3(a) and 3(b)) and is found independent of *S13-S4-S11* in the genomes of Lokiarchaeota, Heimdallarchaeota, and three Thorarchaeotes. The gene for the DNA-directed RNA polymerase subunit N (*rpoN*), when not found in the main cluster, is often associated with the *L7ae* cluster. In two Asgard Thorarchaeote genomes, part of the *L18e* cluster cooccurs with the *str/L30e* cluster. In all the Asgard Thorarchaeote genomes examined, a partial portion of the *L7ae* cluster cooccurs with either a part of or the whole of the *rpoD-L18e-L13-S9* segment.

### 3.5. S24e-S27ae Cluster

This cluster has a core of eight genes (*gcp*-*pNP*-*S15-S3ae-S27ae-S24e*-*rpoE2-rpoE1*). It cooccurs and is contiguous with the *L7ae* and the *Alpha-L18e* clusters in the Asgard Odinarchaeota (Figure 4(b)). *gcp* and *pNP* encode a bifunctional tRNA threonylcarbamoyladenosine biosynthesis protein and a noncanonical purine NTP pyrophosphatase, respectively.

Based on how it is found in the rest of the archaeal genomes, the *S24e* cluster can be split into two sections, one containing the three genes, *pNP-S15-S3ae*, and the other with five genes, *rpoE1-rpoE2-S24e-S27ae*-*gcp*. In all five complete Lokiarchaeote genomes, the *pNP-S15-S3ae* section occurs independently. In three of the Lokiarchaeote genomes, the second segment cooccurs with a part of the *L7ae* cluster. A section comprising *S27ae-S24e-rpoE2-rpoE1* cluster cooccurs with the *S6e-eIF2g-utp24* section of *L7ae* cluster in all the Asgard Thorarchaeota examined. In the two complete Asgard Heimdallarchaeota (PR6 and PM71), the two sections of the *S24e* cluster “sandwich” a small segment of the *L7ae* cluster, viz., *S6e* and *Utp24-eIF2g*. In four Heimdallarchaeotes, the *pNP-S15-S3ae* section is found in the immediate neighborhood of one section of the *spc* cluster. Amongst the non-Asgard Archaea, the *S24e-S27ae-rpoE1* cluster is contiguous with the *L7ae* cluster, the *Alpha-L18e*, and the *str/L30e* clusters in the Desulfurococcales (Crenarchaeota) Archaea, namely, *D. amylolyticus* 1221n and *S. hellenicus* DSM 12710 (Supplementary Figure 2).

### 3.6. Identification of a New Cluster of r-protein Coding Genes

A new cluster *L7ae* comprised of eight genes (*L7ae-S28e-L24e-ndk-infB-S6e-eIF2g-*Utp24) was identified in the Asgard Odinarchaeota (Figure 4). The genes *ndk*, *infB*, *eIF2g*, and *Utp24* encode a nucleoside diphosphate kinase, a translation initiation factor IF-2, the gamma subunit of translation initiation factor 2, and a 30S proteasome protein, respectively. In the Odinarchaeote genome, this cluster co-occurs with the entire *S24e-S27ae* cluster, in the following order: *S24e-S27ae* cluster, *L7ae* cluster, archaeal mevalonate pathway genes [81] and a partial *L18e* cluster. Such a contiguous arrangement of multiple clusters was also found in the genomes of *D. amylolyticus* 1221n and *S. hellenicus* DSM 12710 (Desulfurococcales (Crenarchaeota)) (Supplementary Figure 2). In three Lokiarchaeote genomes, *L7ae..infB* (5 genes, since *ndk* could not be found in these genomes using a BLAST search) cooccur with a portion of the *S24e-S27ae* cluster. In the two complete genomes of Heimdallarchaeotes PR6 and PM71, 3 genes, *S6e-eIF2g-utp24*, are sandwiched in between two parts of the *S24e-S27ae* cluster (Figure 4). In the two complete Asgard Thorarchaeote genomes, this 3-gene section *S6e-eIF2g-utp24* cooccurs with a five-gene segment of the *S24e-S27ae* cluster. In the other two Thorarchaeote genomes, a partial portion (5 genes) of this new cluster is found sandwiched between the *str/L30e* and a partial section (6 genes) of the *L18e* clusters.

### 3.7. *L31e* and *L11-L1-L10-L12* Clusters

The genes for the Archaea unique r-proteins, namely, L39e, L31e, S19e, and LXa, have been described to be part of a cluster that includes the conserved segment of 4 genes, viz., *S19e-COG2118-COG2117-L39e* [20]. This 4-gene conserved segment occurs as such only in the non-Asgard Archaea *M. jannaschii*. COG2117 is missing in all the other non-Asgard and Asgard Archaea examined (Figure 5). In the Asgard genomes of Odinarchaeota LCB4 and the three Thorarchaeote strains, this segment is part of a larger contiguous segment that includes the four gene *L11-L1-L10-L12* cluster, which is described as an operon [82, 83]. The genes in this cluster include those encoding RNP (ribonuclease P protein component 4), RBP (an RNA binding protein), Nep1 (ribosomal RNA small subunit methyltransferase), Spt5 (transcription elongation factor), SecE (gamma subunit of the protein translocase SEC61 complex), RbsK (a carbohydrate kinase), FtsZ (a cell division protein), FtsY (a signal recognition particle-docking protein), and DtdA (D-tyrosyl-tRNA(Tyr) deacylase).

In the Asgard genomes examined in this study, except four Asgard genomes, all the other genomes show a conserved 5-gene core cluster, *S19e-COG2118-L39e-L31e* (Figures 5(a) and 5(b)). The remaining genes of the *L31e* and *L11-L1-L10-L12* clusters show significant rearrangements in the Asgard Archaea. Overall, it can be divided into four tentative segments, viz., *nep1-RNP-RBP-S19e-COG2118-L39e-L31e-eIF6*, *LXa-pfdA-ftsY*, *ftsZ-secE-spt5*, and *L11-L1-L10-L12*.

In the Asgard Lokiarchaeotes MKD1 and FW102, part of the large segment, *S19e..eIF6*, is contiguous with *LXa…secE*, and in B-35, bin132, and bin108, *nep1…secE* occurs as a whole unit. The *L11-L1-L10-L12* segment is on a different location in these genomes. In all the Asgard Heimdallarchaeotes examined, *L11* is split from its operon/cluster (*L11-L1-L10-L12*) and found in other parts of the cluster. In the two complete Heimdallarchaeotes PR6 and PM71, the remainder of the operon *L12-L10-L1* while on the negative strand is contiguous with the section containing *S19e..eIF6* on the positive strand, followed by *RBP..ftsZ* on the negative strand. The three-gene segment *ftsY-pfdA-LXa* occurs independently. This pattern of rearrangement is identical in these two Asgard Heimdallarchaeotes, with the only difference being the strand orientation of all these segments is the opposite relative to each other. In the incomplete Heimdallarchaeote bin2.246 genome (16 contigs) and three Heimdallarchaeote genomes (bin6, bin272, and bin76), parts of these segments occur as *L11*, contiguous with *L39e-L31e-eIF6-LXa-ftsY*, followed by *L10-spt5-secE*. These three genomes also show truncated versions of the *S10-spc* cluster (Figure 1).

Additionally, an annotation error was observed in the Asgard Thorarchaeote BC genome. A hypothetical protein coding gene (GenBank accession number: TXT56565.1) is downstream of *rbsK* (GenBank accession number: TXT56564.1, which is also incorrectly annotated as a hypothetical protein). This gene is likely an artifact of erroneous annotation, because the coordinates of the same (gene for TXT56565.1: 125555-125674) overlap the coordinates of the gene *ftsZ* immediately downstream by 28 bases (gene for TXT56566.1: 125646-126779). Overall, the *S19e-COG2118-L39e* order is conserved in all the genomes with the exception of the non-Asgard Archaea Thaumarchaeota (Figure 5).

## 4. Discussion

Classical operons are found to occur in both Bacteria and Archaea [84, 85]. The conservation of the arrangement of the r-protein gene cluster is in congruence with how they are conserved in sequence [21, 22, 55]. A large chunk of 14 genes from the most highly conserved *S10-spc* cluster of r-protein coding genes was thus found to be similarly conserved in arrangement, whether it was Bacteria, Asgard, or non-Asgard Archaea. The arrangement of the four r-protein coding gene clusters as observed in the 17 Asgard archaeal genomes (Lokiarchaeota, Odinarchaeota, Heimdallarchaeota, and Thorachaeota) suggests that apart from the big cluster (*S10-spc*), the arrangement/order of the rest of the r-protein clusters is not conserved and is dispersed. In the context of one section of the *Alpha* operon found in the vicinity of the *S10-spc* cluster, the conservation of the location resembles what is observed in bacterial genomes. Assuming these Asgard genomes have been correctly assembled, a portion of the *Alpha* operon (*L18e*) cluster associated with the highly conserved and universal *S10-spc* cluster is likely a reflection of their association despite the phylogenetic distance between Bacteria and Asgard Archaea.

It has been suggested that the Heimdallarchaeota group is the most likely sister group of Eukarya [7, 36, 40, 43, 44]. The dispersive nature of the *str/L30e* cluster order and the unique arrangement of the *S24e-S27ae*, the *L7ae*, the *L31e*, and the *L12-L10-L11-L1* clusters are particularly evident in the case of the Heimdallarchaeote genomes. The unique arrangement of the r-protein clusters, as observed in the case of the Heimdallarchaeota members of the Asgard in this study, may corroborate this. This could possibly be a marker for evolutionary divergence within the Asgard genomes. Additional complete Asgard genomes, when available, may help understand such divergences better. Examination of the plastid genome of *A. thaliana* and the gene order of (*S10-spc* cluster)-*L36-S11* shows similarities with the order in Bacteria (*E. coli*, *B. subtilis*), though a section of this cluster is missing from the same (data not shown).

The dispersion of gene clusters in eukaryotes has been suggested to be directly correlated with higher rates of chromosomal/genome evolution [26, 86]. It remains to be seen if Asgard Archaea show a higher rate of genome evolution as compared to the non-Asgard Archaea.

Genome organization seldom remains in stasis with the phenomenon of lateral gene transfer constantly changing the dynamics of the same. It has been hypothesized that the Asgard proteome, particularly the eukaryotic signature protein coding genes, evolved through extensive horizontal gene transfer [36]. It is not clear from the few available complete Asgard genomes if the arrangement of the r-protein clusters is a result of this phenomenon. Nevertheless, the roles of gene order or arrangement in fashioning cellular physiology and genome evolution remain to be explored further [87].

It should be noted that the arrangement of the r-protein coding gene clusters in the Asgard could be in some cases an artifact of poor genome completion. The occurrence of several partial genes, a universal gene annotated as a pseudogene, and several universal gene segments missing in the Asgard genomes in this study (Figures 1–5) suggests that the genomes in which they are found may have been incorrectly assembled. The noncontiguous/disjointed occurrence of the otherwise conserved segment of 14 cooccurring genes of the *S10-spc* cluster in the three Heimdallarchaeotes (bin6, bin272, and bin76) (Figure 1(a)) and the absence of three of the most universal and highly conserved genes *uL3-uL4-uL23* from the genomes of Lokiarchaeotes bin132 and bin108 (Figure 1) suggest that the assembly of these genomes is likely not accurate. The incompleteness or misassembly of genomic contigs has been observed previously [88–92]. In fact, binning errors were implicated in the absence of r-protein genes from metagenome-assembled genomes (MAGs), and hence, analysis of genomics and phylogeny of uncultivated microbes, using MAGs, may be fraught with erroneous interpretations [93, 94].

However, we did observe one interesting feature in a non-Asgard archaeal MAG. Of the three non-Asgard archaeal genomes assembled from metagenomes that are available in the NCBI database [88], only the genome of DPANN archaeon GW2011_AR10 (GenBank accession: CP010424) is complete as a single scaffold/chromosome. Examining the order/arrangement of the r-protein gene clusters in this genome showed that four of the five clusters reported in this paper cooccur contiguously. The arrangement of these clusters in this genome is as follows: *str-L30e* cluster (*rpoH-rpoB-HP-rpoA1-rpoA2-L30e-nusA-S12-S7-fusA-EF(1a)-S10*)*-5HPs*-*S10-spc* cluster (*L3-L4-L23-L2-S19-L22-S3-L29-IF(Sui)-RNP1-S17-L14-L24-S4e-L5-S14-S8-L6-L32e-L19e-L18-S5-L30-L15-secY*)-*HP-L34e-L14e-L18e* (*Alpha operon*) cluster (*S13-S4-S11-rpoD-tRNA(leu)-L18e-L13-S9-rpoN-S2*)-HP-gltX-HP-L7ae cluster (*L7ae-S28e-L24e-ndk-dUTP diphosphatase-infB-S6e-eIF2g-Utp24-rpoE1-rpoE2-S24e-S27ae*-10 ORFS-*gcp-S15-HP-S3ae*). The *L31e* and *L11-L1-L10-L12* clusters are not part of this section and occur separately as two distinct sections elsewhere in the genome. Only in *B. subtilis* (Bacteria), a partially similar feature is observed with the *L11* cluster found immediately upstream of the *S10*-*spc* cluster.

The second non-Asgard MAG, that of DPANN *Candidatus Micrarchaeum acidiphilum* ARMAN-2 (GenBank assembly: GCA_009387755.1) is in 8 contigs, with the r-protein gene clusters split and not conserved as reported for some of the Asgard genomes in the paper. The third non-Asgard MAG, that of DPANN *Candidatus Forterrea multitransposorum* archaeon (GenBank accession no.: CP045477), has not been annotated into ORFs (open reading frames)/genes etc. Thus, these sequences derived from metagenome-assembled genomes may not be sound.

It is not clear if the cooccurrence of four r-protein clusters in the MAG of DPANN archaeon GW2011_AR10 is a result of the accuracy of the assembly and annotation/curation of the genome. If it is indeed an accurate assemble, then the assembly of other genomes in the database may need to be revisited for improving better quality which may help in an improved understanding of arrangement of these clusters. Thus, in the absence of good quality genomes, ascertaining the order of highly conserved genes of r-proteins can be challenging, and thus, well-curated genomes are vital towards understanding genome organization. The importance of a more robust curation of MAGS as well publicly available Asgard genomes [6, 95] cannot be emphasized enough to get a better picture of the genome organization of the same.

## 5. Conclusions

The discovery of the Asgard Archaea has redefined our understanding of the phylogenetic positions of the various groups that comprise the Archaea. The genomic arrangement of the r-protein coding genes in the Asgard superphylum within the domain Archaea suggests that the order may follow the phylogeny. The closer an Asgard is to eukaryotes, the more dispersed is the arrangement of the r-protein gene clusters. This feature suggests a possible ancient gain or loss of the operon strategy of gene regulation in the early history of life. Given that very few Asgard genomes are currently complete, new data and better means of genome assembly will facilitate a better understanding of genome order in the Asgard Archaea in the future.

## Figures and Tables

**Table 1:** List of Asgard genomes used in this study.

*Archaea* (*Asgard* group)	Accession number	No. of contigs	Assembly	GenBank sequence/INSDC
Lokiarchaeota				
MK-D1	GCA_008000775.1	^∗^	ASM800077v1	CP042905.1
*H. repetitus* FW102	GCA_021498095.1	^∗^	ASM2149809v1	JAIZWK010000001.1
B-35	GCA_025839675.1	^∗^	ASM2583967v1	CP104013.1
bin132	GCA_020343655.1	^∗^	ASM2034365v1	CP070805.1
bin108	GCA_020344955.1	^∗^	ASM2034495v1	CP070831.1
Odinarchaeota LCB_4	GCA_001940665.1	^∗^	ASM194066v1	MDVT00000000.1
Thorarchaeota				
bin27	GCA_020348985.1	^∗^	ASM2034898v1	CP070761.1
bin8	GCA_020355105.1	^∗^	ASM2035510v1	CP070658.1
FW25	GCA_021498125.1	12	ASM2149812v1	JAIZWL01
BC	GCA_008080745.1	19	ASM808074v1	SHMX01
Heimdallarchaeota				
PM71	GCA_021513695.1	^∗^	ASM2151369v1	CP084166.1
PR6	GCA_021513715.1	^∗^	ASM2151371v1	CP084167.1
bin6	GCA_020353515.1	^∗^	ASM2035351v1	CP070695.1
bin76	GCA_020351745.1	^∗^	ASM2035174v1	CP070665.1
bin272	GCA_020348965.1	^∗^	ASM2034896v1	CP070760.1
AC18	GCA_021498085.1	11	ASM2149808v1	JAIZWM01
SZ_4_bin2.246	GCA_011364965.1	16	ASM1136496v1	RDOB01

^∗^Complete genome.

## Data Availability

The datasets used and analysed within the current study are available from the NCBI website as referenced in the paper.

## Abbreviations

MKD1: Lokiarchaeote strain *Candidatus Prometheoarchaeum syntrophicum* MK-D1
FW102: Lokiarchaeote strain *Harpocratesius repetitus* FW102
B-35: Lokiarchaeote strain B-35
bin132: Lokiarchaeote strain bin132
bin108: Lokiarchaeote strain bin108
LCB4: Odinarchaeote strain LCB_4
bin27: Thorarchaeote bin27
bin8: Thorarchaeote bin8
FW25: Thorarchaeote strain FW25
BC: Thorarchaeote strain BC
PM71: Heimdallarchaeote strain PM71
PR6: Heimdallarchaeote strain PR6
bin6: Heimdallarchaeote bin6
bin76: Heimdallarchaeote bin76
bin272: Heimdallarchaeote bin272
AC18: Heimdallarchaeote AC18
bin2.246: Heimdallarchaeote SZ_4_bin2.246
LUCA: Last universal common ancestor
LSU: Large subunit
SSU: Small subunit
r-protein: Ribosomal protein
mtDNA: Mitochondrial DNA
NCBI: National Center for Biotechnology Information
rRNA: Ribosomal RNA
MAGs: Metagenome-assembled genomes
IMG/M: Integrated Microbial Genomes and Microbiomes
DOE: Department of Energy
JGI: Joint Genome Institute.

## Supplementary Materials

Supplementary Materials Supplementary Figure 1: (a) the *S10-spc* cluster distributed in multiple contigs in Asgard Archaea. In *Odinarchaeota* LCB_4, arrows represent different locations on a single contig; adjacent arrows do not indicate their order on the genome; genes within an arrow are contiguous; the direction of the arrow denotes the strand orientation. ^∗^Begins or ends a contig. Hsp20: heat shock protein 20 (a, partial gene; d, pseudogene). Red diamond represents gene(s) absent in the corresponding genome/location; arrows within dashed red boxes are contiguous. HP: hypothetical protein; RNP1: ribonuclease P protein component 1; NCG: noncluster genes. ^#^ORF(s) annotated as HP(s). Hsp20: heat shock protein 20. Blank box represents the entire gene set missing in corresponding genome location. (b) The *S10-spc* cluster distributed in multiple contigs in Asgard Archaea. Adjacent arrows do not indicate their order on the genome; genes within an arrow are contiguous; the direction of the arrow denotes the strand orientation. ^∗^Begins or ends a contig. Hsp20: heat shock protein 20 (a, partial gene; d, pseudogene). Red diamond represents gene(s) absent in the corresponding genome/location; arrows within dashed red boxes are contiguous. HP: hypothetical protein; RNP1: ribonuclease P protein component 1; NCG: noncluster gene(s). ^#^ORF(s) annotated as HP(s). Hsp20: heat shock protein 20. Blank box represents the entire gene set missing in corresponding genome location. Supplementary Figure 2: the *S24e-S27ae-rpoE1* cluster is contiguous with *L7ae…Utp24*, the *Alpha-L18e*, and the *str/L30e* clusters in the Desulfurococcales (Crenarchaeota) Archaea, namely, *D. amylolyticus* 1221n and *S. hellenicus* DSM 12710. Genes within an arrow are contiguous; red diamond represents gene(s) absent in the corresponding genome/location. ORFs: open reading frames. Arrows within dashed red boxes are contiguous. HP: hypothetical protein.
