# Supplementary material for: Ribosomal Protein Cluster Organization in Asgard Archaea
Source: Archaea. 2023 Sep 29;2023:5512414. doi: 10.1155/2023/5512414 (PMC10833476; doi:10.1155/2023/5512414)
Supplement: Supplementary Materials — Supplementary Figure 1: (a) the S10-spc cluster distributed in multiple contigs in Asgard Archaea. In Odinarchaeota LCB_4, arrows represent different locations on a single contig; adjacent arrows do not indicate their order on the genome; genes within an arrow are contiguous; the direction of the arrow denotes the strand orientation. ∗Begins or ends a contig. Hsp20: heat shock protein 20 (a, partial gene; d, pseudogene). Red diamond represents gene(s) absent in the corresponding genome/location; arrows within dashed red boxes are contiguous. HP: hypothetical protein; RNP1: ribonuclease P protein component 1; NCG: noncluster genes. #ORF(s) annotated as HP(s). Hsp20: heat shock protein 20. Blank box represents the entire gene set missing in corresponding genome location. (b) The S10-spc cluster distributed in multiple contigs in Asgard Archaea. Adjacent arrows do not indicate their order on the genome; genes within an arrow are contiguous; the direction of the arrow denotes the strand orientation. ∗Begins or ends a contig. Hsp20: heat shock protein 20 (a, partial gene; d, pseudogene). Red diamond represents gene(s) absent in the corresponding genome/location; arrows within dashed red boxes are contiguous. HP: hypothetical protein; RNP1: ribonuclease P protein component 1; NCG: noncluster gene(s). #ORF(s) annotated as HP(s). Hsp20: heat shock protein 20. Blank box represents the entire gene set missing in corresponding genome location. Supplementary Figure 2: the S24e-S27ae-rpoE1 cluster is contiguous with L7ae…Utp24, the Alpha-L18e, and the str/L30e clusters in the Desulfurococcales (Crenarchaeota) Archaea, namely, D. amylolyticus 1221n and S. hellenicus DSM 12710. Genes within an arrow are contiguous; red diamond represents gene(s) absent in the corresponding genome/location. ORFs: open reading frames. Arrows within dashed red boxes are contiguous. HP: hypothetical protein. [file 5512414.f1.pdf]

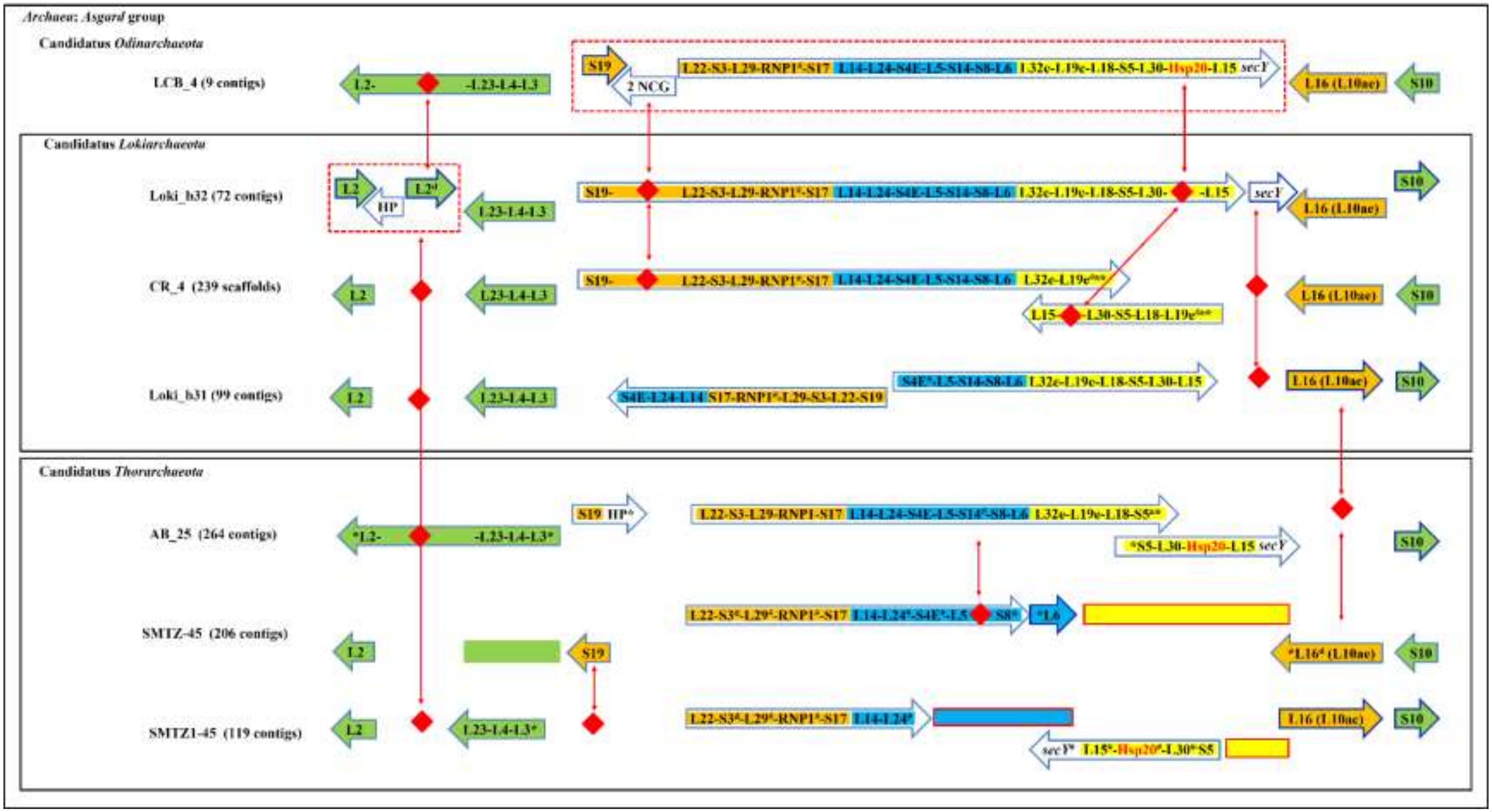

Supplementary Figure 1a: The *S10-spc* cluster distributed in multiple contigs in Asgard archaea. In *Odinarchaeota* LCB_4, arrows represent different locations on a single contig; adjacent arrows do not indicate their order on the genome; genes within an arrow are contiguous; the direction of the arrow denotes the strand orientation; * begins or ends a contig; Hsp20 – Heat shock protein 20: a- partial gene; d - pseudogene; ◆ gene(s) absent in the corresponding genome/location; arrows within dashed RED boxes are contiguous; HP- hypothetical protein; RNP1 - Ribonuclease P protein component 1; NCG - non cluster genes; # -ORF(s) annotated as HP(s); Hsp20 – Heat shock protein 20; blank box – entire gene set missing in corresponding genome location.

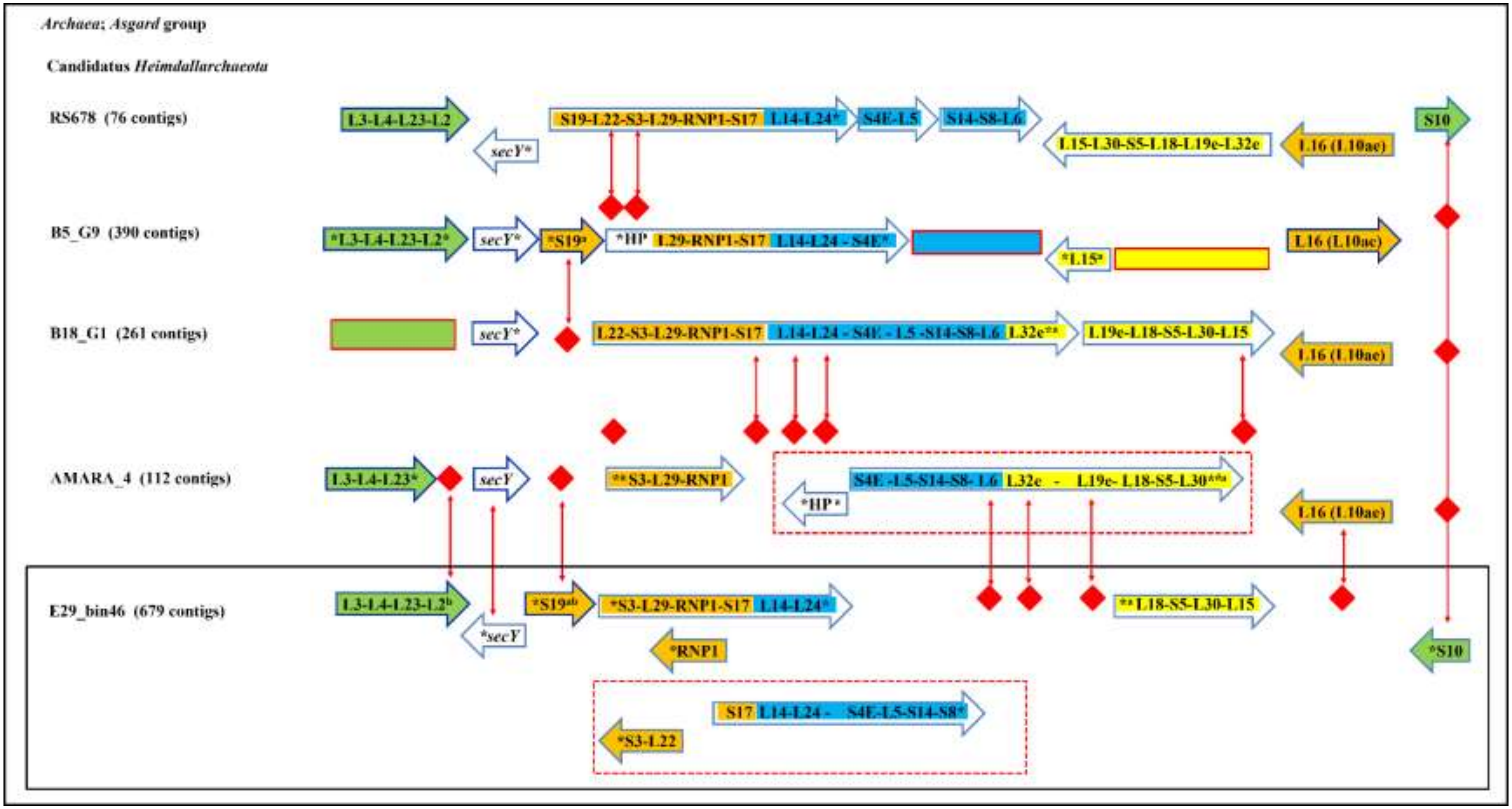

Supplementary Figure 1b: The *S10-spc* cluster distributed in multiple contigs in Asgard archaea. Adjacent arrows do not indicate their order on the genome; genes within an arrow are contiguous; the direction of the arrow denotes the strand orientation; * begins or ends a contig; Hsp20 – Heat shock protein 20: a - partial gene; NCG - non-cluster gene(s); d- pseudogene; ◆ gene(s) absent in the corresponding genome/location; arrows within dashed RED boxes are contiguous; HP- hypothetical protein; RNP1 - Ribonuclease P protein component 1; NCG - non cluster gene(s); # -ORF(s) annotated as HP(s); Hsp20 – Heat shock protein 20; blank box – entire gene set missing in corresponding genome location.

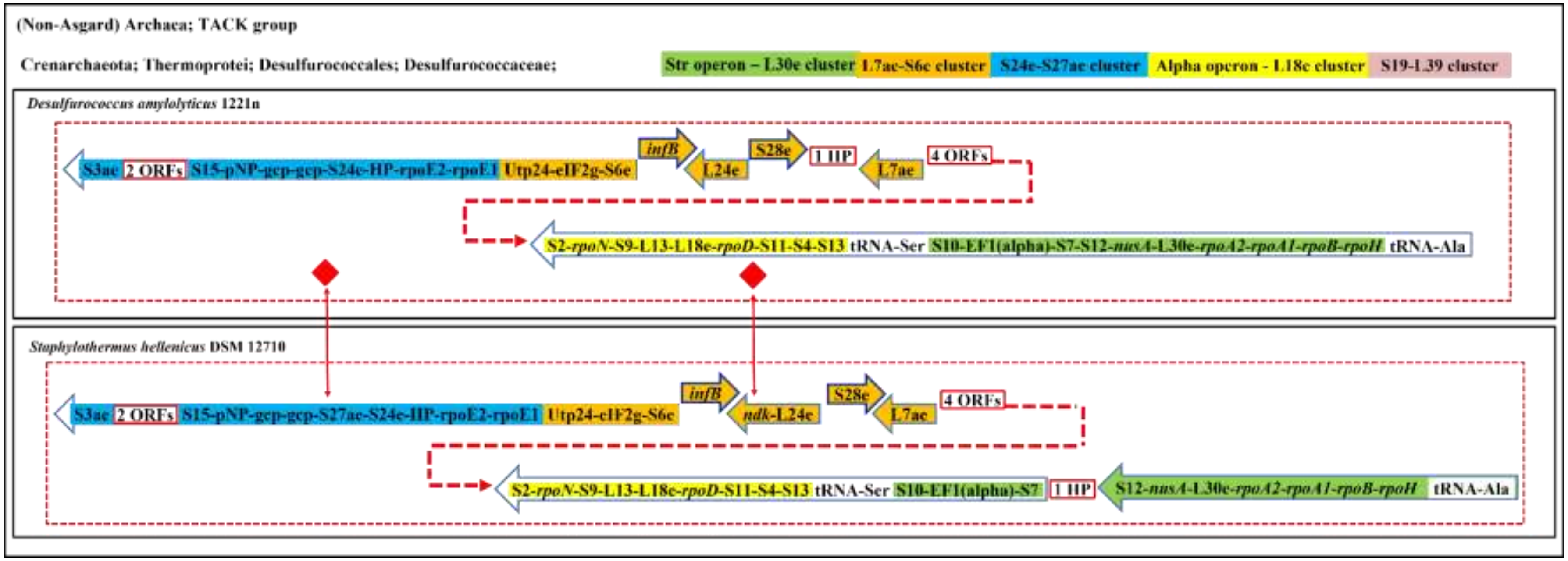

Supplementary Figure 2: The *S24e-S27ae-rpoE1*cluster is contiguous with *L7ae…Utp24*, the *Alpha-L18e* and the *str/L30e* clusters in the Desulfurococcales (Crenarchaeota) archaea, namely, *D. amylolyticus* 1221n and *S. hellenicus* DSM 12710. Genes within an arrow are contiguous; ◆ gene(s) absent in the corresponding genome/location; ORFs - Open reading frames; arrows within dashed RED boxes are contiguous; HP- hypothetical protein.
